# MicroRNAs as potential target in human bone and soft tissue sarcoma therapeutics

**DOI:** 10.3389/fmolb.2015.00031

**Published:** 2015-06-17

**Authors:** Jyotika Varshney, Subbaya Subramanian

**Affiliations:** ^1^Department of Surgery, University of MinnesotaMinneapolis, MN, USA; ^2^Masonic Cancer Center, University of MinnesotaMinneapolis, MN, USA

**Keywords:** sarcoma, microRNAs, osteosarcoma, soft tissue sarcoma, therapeutics, pediatric oncology, bone sarcoma

## Abstract

Sarcomas are highly aggressive heterogeneous tumors that are mesenchymal in origin. There have been vast advancements on identifying diagnostic markers for sarcomas including chromosomal translocations, but very little progress has been made to identify targeted therapies against them. The tumor heterogeneity, genetic complexity and the lack of drug studies make it challenging to recognize the potential targets and also accounts for the inadequate treatments in sarcomas. In recent years, microRNAs that are a part of small non-coding RNAs have shown promising results as potential diagnostic and prognostic biomarkers in multiple sarcoma types. This review focuses on the current knowledge of the microRNAs that are deregulated in sarcomas, and an insight on the strategies to target these microRNAs that are essential for developing improved therapies for various human sarcomas.

## Introduction

Sarcomas are malignant tumors that are mesenchymal in origin (Taylor et al., 2011a). They are rare, highly complex heterogeneous tumors accounting for about 1% of all other tumor types in humans. They are of particular importance to pediatrics as 15% of all pediatric malignant tumors are sarcomas. Sarcomas are known to occur in different body parts and based on the cell/tissue type can be broadly identified as bone or soft tissue sarcomas and further classified into more than 50 subtypes (Weiss et al., 2007). Additionally, there is another set of classifications that are based on the genetic alterations (Stephens et al., 2011). The first category is based on genetic alterations that include translocations or specific mutations. Most of the sarcomas fall under this first category. However there are some sarcomas such as leiomyosarcoma and osteosarcoma, that fall into a second category that present complex karyotypes with multiple genomic instabilities.

The general standard of care for most sarcoma types consists of surgical resection and chemotherapy. This method yields poor responses and the majority of cases succumb due to lung metastasis. Despite the advancements in diagnosis and prognosis of the disease, treatment options have remained stagnant for the past three decades. Potential reasons for this include rarity of tumor incidence, genetic complexity, and late stage at diagnosis leading to poor prognosis. All the above reasons lead to an urgent need to identify novel biomarkers that are targetable, which will eventually lead to better diagnosis and prognosis of sarcomas.

MicroRNAs (miRNAs) have a crucial role in predicting the disease outcome and the prognosis in various cancer types including sarcoma (Calin et al., 2008; Cuiffo et al., 2014). miRNAs are a group of small non-coding RNAs that regulate genes post transcriptionally. Over the past few years, various miRNA deregulations have been accounted an aid in histological classification and clinical relevance of sarcomas. To address the role of miRNAs in various sarcomas, this review will highlight how various miRNAs play crucial roles in human sarcomas and an insight on current miRNA target based therapeutic approaches.

## Bone sarcomas

Bones sarcoma consists of only 0.2% of all the neoplasms. Major known bone sarcomas include osteosarcoma, chondrosarcoma, and Ewing sarcoma (Burningham et al., 2012). The sub-classification of these bone sarcomas is based on their histology, cell of origin and the tissue site. We will discuss each of the bone sarcomas below.

## Osteosarcoma

Osteosarcoma is the most common primary malignant bone tumor and primarily occurs in adolescents. The incidence rate of osteosarcoma is approximately 4–5 cases per million (Mirabello et al., 2009). It usually arises from the metaphysis of the long bones such as the femur. Studies, including ours, have shown that miRNAs deregulation occurs in osteosarcoma (Maire et al., 2011; Thayanithy et al., 2012). We observed that there is a significant downregulation of around 50 miRNAs at the chromosome 14q32 locus (14q32 miRNAs) in osteosarcoma compared to the normal bone without any alterations in the copy number changes at the locus (Thayanithy et al., 2012). This suggests that there might be additional epigenetic changes in that region. The bioinformatics predictions identified a subset of these 50 miRNAs (miR-382, miR-369-5p, miR-544, and miR-134) that could potentially target the 3′UTR of the *cMYC* transcript (Thayanithy et al., 2012). Upon functional validation, we found that restoring the expression of the four 14q32 miRNAs decrease the cMYC levels and induced apoptosis in osteosarcoma cell lines (Saos2). In the same set of cell lines the 14q32 miRNAs overexpression led to significant decrease in an oncogenic miRNA cluster called miR-17-92, which also happens to be a transcriptional target of *cMYC* (Olive et al., 2010). We also performed rescue experiments where we both overexpressed cMYC in absence of its 3′UTR, or the miR-17-92 cluster and found that osteosarcoma cells could be rescued from the pro-apoptotic effects of the 14q32 miRNAs. In total, we observed that there is a deregulation that involves 14q32 miRNAs, cMYC and miR-17-92, which could contribute to osteosarcoma progression (Maire et al., 2011; Thayanithy et al., 2012). Maire et al. also found that there is deregulation of miRNAs that could target multiple signaling pathways such as MAPK, Wnt and RAS/p21 that played a crucial role in osteosarcoma. In order to find the relationship between various miRNAs and pathways, they generated a comprehensive genetic map that integrated miRNAs and gene expression profiles obtained from various osteosarcoma tumor samples. The mentioned authors, in concurrence with our observation, found the deregulation in miR-382 and cMYC levels in osteosarcoma, further suggesting the role of miRNAs in osteosarcoma progression.

There are several other groups that have contributed in understanding the role of miRNAs in osteosarcoma (Duan et al., 2011; Lulla et al., 2011; Jones et al., 2012). Braun et al. found that miRNAs such as miR-192 and miR-215 are p53 responsive miRNAs that are capable of causing cell cycle arrest in the osteosarcoma cell line U2OS that carries a wild-type p53 (Braun et al., 2008). Other groups have also shown that miRNAs such as miR-34a and miR-31 could arrest proliferation in osteosarcoma cell lines in association with p53 (Creighton et al., 2010; Yan et al., 2012). miR-31 was also shown to target multiple metastatic genes such as integrin A5, radixin, and RhoA indicating its potential role in the prevention of metastasis in osteosarcoma (Creighton et al., 2010).

There are several studies that showed that miRNAs might have a potential therapeutic role in osteosarcoma as well. For instance Duan et al. found that miR-199a-3p restoration decreased mTOR and Signal Transducer and Activator of Transcription (STAT) expression and proliferation and migration in osteosarcoma cells making it a strong therapeutic candidate in osteosarcoma (Duan et al., 2011). Similar results were obtained with knock down of miR-21 that is usually overexpressed in osteosarcoma, which targets RECK and affects the activation of MMPs (Kang et al., 2007; Ziyan et al., 2011). Another miRNA, miR-183 targets Ezrin that leads to suppression of migration and invasion in osteosarcoma cells. Further, it has been shown that miR-183 correlates with pulmonary metastasis and local recurrence of osteosarcoma, suggesting its crucial role in metastasis (Zhu et al., 2012).

miRNAs have also been shown to be useful as biomarkers of chemotherapeutic responses in osteosarcoma. Some of the first known miRNAs that have been associated with drug sensitivity are miR-140 and miR-215 (Song et al., 2009, 2010; Zhu et al., 2012). The studies revealed that miR-140 overexpression led to chemoresistance to various chemotherapeutics such as methotrexate (MTX) and 5- fluorouracil (5-FU). Also, miR-140 negatively regulated histone deacetylase 4 (HDAC 4) resulting in 5-FU resistance. Similarly, miR-215 induced chemoresistance to MTX in U2OS cells. Another study examined the miRNA expression of 27 paraffin embedded osteosarcoma samples to determine chemoresistance to ifosfamide (IFO) (Gougelet et al., 2011). They performed supervised hierarchical clustering and found five miRNAs, miR-92a, miR-99b, miR-132, miR-193-5p, and miR-422 that could significantly differentiate between good and poor responders to IFO. A recent study by Lauvrak et al. evaluated miRNAs in 22 osoteosarcoma cell lines that are crucial for various cellular functions including proliferation, invasion, and migration (Lauvrak et al., 2013). They found that miR-199b-5p and miR-100-3p were downregulated and miR-155-5p, miR-135-5p, and miR-146-5p upregulated in more aggressive osteosarcoma cell lines. Further studies would be required to correlate these miRNAs with genes that regulate tumor growth and metastasis in osteosarcoma. Thus, such studies unveil the multiple roles of miRNAs in prognosis, diagnosis, therapeutics, and drug resistance in osteosarcoma and other sarcomas.

## Ewing sarcoma

Ewing sarcoma is a pediatric sarcoma that usually originates in bone. The most common genetic alteration is transcript fusion of transcription regulators EWS and Ets leading to a novel transcriptional factor that drives oncogenesis. Two independent studies revealed global miRNA expression changes due to stable repression of fusion proteins in Ewing sarcoma (Ban et al., 2011; McKinsey et al., 2011; Dylla et al., 2013). The miRNAs predicted in one of these studies showed that these miRNAs could potentially target insulin-like growth factor (IGF), which is a crucial driver of Ewing sarcomagenesis (McKinsey et al., 2011). The other study identified miR-145 as a top candidate miRNA that is repressed in Ewing sarcoma (Ban et al., 2011). Interestingly, miR-145 could directly target the EWS/Fli1 fusion transcript and this regulatory network should be explored further as a potential miRNA-mediated therapy in this type of cancer. Let-7a is another miRNA that was identified as a direct target toward the fusion transcript (De Vito et al., 2011).

Other studies involved investigating the role of miRNAs as biomarkers in Ewing's sarcoma (Mosakhani et al., 2012; Nakatani et al., 2012). One of the groups showed that patients with high levels of miR-34a had better 5-year survival outcome than patients with low levels of miR-34a (Nakatani et al., 2012). In an integrated analysis study of miRNAs and copy number changes revealed around 20 differentially expressed miRNAs in chromosomal regions with altered copy numbers (Mosakhani et al., 2012). A recent study identified 35 miRNAs that were differentially expressed in Ewing sarcoma tissues and cell lines compared to the mesenchymal stem cells (Karnuth et al., 2014). They identified miR-31 as a potential tumor suppressor that affected proliferation and invasion in Ewing cell lines. Interestingly, the group found no significant differentially expressed miRNAs that could possibly distinguish between Ewing sarcoma samples that were from primary and metastatic group.

## Chondrosarcoma

Chondrosarcoma is a rare malignant tumor arising from transformed cartilaginous cells. This happens when osteochondromas transform into chondrosarcoma and usually occurs in 1–5% of patients. Several studies have shown involvement of miRNAs in chondrogenesis and cartilage based diseases (Tuddenham et al., 2006; Lin et al., 2009; Zhang et al., 2013). One study shows that there are eight miRNAs that are deregulated in osteochondroma patients compared to normal subjects (Zuntini et al., 2010). These miRNAs were further shown to regulate genes that are involved in normal growth plate formation that includes heparin sulfate and glycan structure biosynthesis. Hameetman et al. identified a similar differential miRNA profile in chondrosarcoma patients indicating the miRNAs involvement in osteochondroma malignant transformation to chondrosarcoma (Hameetman et al., 2006). The first significant study of miRNAs in chondrosarcoma tissue samples and cell lines revealed downregulation of let-7a, miR-100, miR-136, miR-222, miR-335, and miR-376a compared to normal chondrocytes (Yoshitaka et al., 2013; Nugent, 2014). Another recent study shows that overexpressing proline-rich polypeptide (PRP-1) inhibited mTORC1, which resulted in upregulation of certain tumor suppressor miRNAs (miR-125b, miR-192) and downregulation of oncomiRs (miR-550, miR-589, miR-490-3p) (Galoian et al., 2014). Similarly, another study demonstrated that overexpression of miR-518b decreased the expression of Bcl-2, which is an anti-apoptotic that led to increased expression of Bax, a pro-apoptotic in human chondrosarcoma cell lines (Liang et al., 2014). The above studies indicate that miRNAs are crucial players in chondrosarcoma and further functional validation should be performed to confirm their role.

## Soft tissue sarcomas

Sarcomas that affect connective tissues such as muscle, fat, and blood vessels fall under the soft tissue sarcomas. Such sarcomas account for approximately 1% of all malignant tumors. We will discuss the commonly known soft tissue sarcomas in the section below.

## Gastrointestinal stromal tumor

Gastrointestinal stromal tumor (GIST) is considered the most common tumor of mesenchymal origin arising from interstitial cells of *Cajal* in the gastrointestinal tract. Most of them have *C-KIT* or *PDGFRA* mutations. We found lower levels of miR-221 and miR-222 that target 3′UTR of KIT leading to KIT gene overexpression, enhancing the oncogenicity of the GIST cells (Subramanian et al., 2008). Another group consistently found lower levels of miR-221 and miR-222 in KIT- positive GISTs compared to normal tissues, suggesting that these miRNAs have a therapeutic potential in KIT-positive GISTs (Felli et al., 2005). Another miRNA, miR-494, which is a negative regulator of KIT has been shown to be significantly low in GISTs which indicates a promising therapeutic target in GISTs (Kim et al., 2011).

Two separate research studies found that there is a significant downregulation of ~44 miRNAs in human chromosome 14q32 region in 12 GIST samples (Haller et al., 2010; Kelly et al., 2013). Moreover, they observed these miRNAs were inversely correlated with GIST tumor progression indicating their prognostic significance. Another group found a lower level of miR-196, which is one of the miRNAs from the human 14q32 chromosomal region that was associated with metastasis and poor survival outcomes (Choi et al., 2010). Thus, these studies indicate the crucial role of miRNAs in diagnostics and therapeutics in GIST patients.

## Rhabdomyosarcoma

Rhabdomyosarcoma (RMS) is a skeletal muscle-derived malignant sarcoma. RMS affects approximately 6–8% of pediatric cases. RMS can be classified into two main subtypes, embyonal RMS (ERMS), and alveolar RMS (ARMS) (Weiss et al., 2007). ARMS is considered more aggressive and has poorer outcomes compared to ERMS (Qualman et al., 1998). ARMS is also associated with various chromosomal translocations that result in an oncogenic fusion protein. A common fusion transcript in ARMS is PAX3-FKHR. The role of miRNAs in RMS is more studied in relation to myomiRs as these sarcomas are thought to originate from myogenic precursors (Subramanian et al., 2008). miRNAs such as the miR-1/ -133/ -206 family are known to specifically regulate myogenic differentiation and muscle tissue homeostasis. Such myomiRs form an attractive area to study for therapeutics in RMS.

Our group and others have shown that miRNA expression profiling is a good tool to determine the differences between ERMS and ARMS (Subramanian et al., 2008). For instance myomiRs are significantly downregulated in RMS and gain-of-function experiments demonstrated that overexpression of the myomiRs would inhibit RMS proliferation suggesting their tumor suppressive role in RMS. We showed that miR-1 and miR-206 were significantly downregulated that led to stabilization of PAX3 and cyclin D2 in RMS. In ARMS, *PAX3* fuses with *FKHR* that leads to the loss of 3′UTR of *PAX3* leading to its oncogenic mechanism in ARMS. Further experiments revealed that overexpression myomiRs such as miR-206 accelerated myogenic differentiation and prevented tumor growth in a xenograft mouse model (Taulli et al., 2009; Yan et al., 2009). The other myomiR, miR-335, is overexpressed in ARMS (Yan et al., 2003). miR-335 is located within intron 2 of *PEG1* that plays a role in muscle differentiation. PEG1 is a known downstream target of PAX3 and it is likely that the *PAX3-FKHR* fusion in ARMS might influence the transcription of *PEG1* and miR-335 in ARMS. Another study aimed to identify miRNAs that are responsible for metastasis in ARMS (Armeanu-Ebinger et al., 2012). They found that miR-9, which is associated with metastasis and invasion, is overexpressed in ARMS. On the contrary miR-200c that inhibits migration was downregulated in ARMS. This suggests that miRNAs could be responsible for metastasis and should be explored further as therapeutic targets in ARMS.

There are several other miRNAs that are not myomiRs, which are affected in RMS. miR-29 levels are significantly low in RMS and re-expression leads to cell cycle arrest and differentiation in RMS cell lines (Weiss et al., 2007). Wang et al. demonstrated that there is activation of NF-κb in RMS, which leads to overexpression of transcription factor YY1 (Wang et al., 2008). The overexpression of YY1 leads to interaction with its cofactor EZH2 leading to downregulation of miR-29b/c in RMS cell lines and tumor tissues. Additionally, miR-29 targets E2F7, which is a crucial cell cycle regulator. Ectopic expression of miR-29 leads to G1 arrest and cell death. Not only does miR-29 regulate cell cycle,it has also been implicated to target HDAC4 and affect epigenetic regulation (Li et al., 2009). miR-183 is another non-myomiR miRNA that plays an oncogenic role in RMS (Sarver et al., 2010a). miR-183 overexpression leads to repression of EGR1 which is a known tumor suppressor and a transcription factor (Sarver et al., 2010a). Low levels of EGR1 affect the levels of transcriptional target PTEN. Thus, miR-183 levels play a crucial role in balancing the levels of two major tumor suppressor genes. These studies demonstrate that myomiRs and non-myomiRs play a major role in RMS and further research should be focused on therapeutics that can correct such miRNA deregulation in RMS.

## Malignant peripheral nerve sheath tumor

Malignant peripheral nerve sheath tumors (MPNSTs) are soft tissue sarcoma tumors that occur in patients with neurofibromatosis type 1 disease. Our group identified p53 inactivation in most of the MPNST patients via a genome-wide transcriptome analysis (Subramanian et al., 2010). In the same study we performed miRNA profiling of benign and malignant PNSTs samples that revealed miR-34a being downregulated in most of the MPNSTs samples compared to benign neurofibromas. Also, we found that miR-214 was upregulated in MPNSTs, which is a direct transcriptional target of metastatic gene *TWIST1* (Yang et al., 2004; Lee et al., 2009). miR-214 was also shown to target *PTEN*, indicating a deregulation in the *TWIST1*-miR-214-*PTEN* pathway in MPNSTs. Another group reported miR-10b upregulation in MPNST tumors and cell lines (Chai et al., 2010). They further performed functional validation by inhibiting miR-10b that led to decrease in cell proliferation, migration, and invasion in MPNST cell lines. They revealed that miR-10b inhibition affected Ras signaling and NF1 expression leading to cellular arrest. miR-21 was also shown to target a neoplastic transformation inhibitor, PDCD4, that led to MPNST tumor formation and progression (Itani et al., 2012). Interestingly, miR-21 was found to be over expressed in schwannomas, which are benign tumors consisting of *NF2* mutations. However, upon gene and miRNA profiling of various schwannomas, neurofibromas and MPNST samples, it was found that both miRNAs and various genes associated with neurofibromas and schwannomas clustered separately compared to MPNSTs, indicating that miRNAs could be useful tool to diagnose and stratify MPNSTs from neurofibromas and schwannomas (Subramanian et al., 2010). Recently, a study showed 16 differentially expressed miRNAs in neurofibromas and MPNSTs from patients with NF1 via microarray analysis (Presneau et al., 2013). Of the 16 differentially expressed miRNAs, 14 were downregulated and 2 miRNAs (miR-210 and miR-339-5p) were upregulated. These miRNAs were further validated by qPCR and overexpression experiments using miRNA mimics in the MPNST cell lines. Also, one of the dowregulated miRNAs, miR-29c was predicted to target various genes including *MMP-2* that are responsible for invasion and metastasis. This demonstrated that overexpression of miR-29c reduced invasion in MPNST cell lines, indicating that certain miRNA levels could be manipulated to reduce metastasis in MPNSTs.

## Synovial sarcoma

Synovial sarcoma (SS) is an adolescent sarcoma originating near the joints and represents around 8% of all soft tissue sarcomas. We were the first group to examine miRNA expression profiles in synovial sarcoma (Subramanian et al., 2008). We found that miR-143 levels were significantly low in SS compared to other soft tissue sarcomas such as GIST and LMS and miR-143 targets *ERK5* or *MAPK7*. EKR5 plays a crucial role in cellular growth and proliferation via tyrosine kinase signaling, and lower levels of miR-143 leads to amplified ERK5 levels leading to SS (Esau et al., 2004). miR-143 is also known to target a common fusion transcript; SS18-SSX1 in SS. Our group revealed that miR-183 is a potential oncomiR in SS (Sarver et al., 2010a). The reason is miR-183 is overexpressed in SS and it targets two important tumor suppressor genes, *EGR1* and *PTEN*, leading to their repression and increase cell proliferation. Hisaoka et al. performed global miRNA expression profiles and found that there are 35 miRNAs, which are differentially expressed in SS compared to other sarcomas (Hisaoka et al., 2011). Let-7e, miR-99b and miR-125a-3p were highly upregulated in SS and arise from the same chromosomal loci. These miRNAs targeted HMGA2 and SMARCA5 that led to cellular proliferation. Such miRNA differential studies and further functional validation will aid in better understanding of miRNAs as a diagnostic and therapeutic potential in synovial sarcomas.

## Leiomyosarcoma

Leiomyosarcoma (LMS) is a sarcoma of uterine smooth muscle. We performed a microarray analysis of LMS and normal smooth muscle samples and found that miRNAs such as miR-1 and miR-133a/b, which are regulators of myogenesis, were significantly overexpressed in LMS (Chen et al., 2006; Subramanian et al., 2008). Another group showed that there are more than 70 miRNAs that are differentially expressed including miR-17-92 in LMS compared to normal uterine smooth muscles (Danielson et al., 2010). Shi et al. demonstrated the association between let-7 and its target HMGA2 (Shi et al., 2009). They functionally validated that lower levels of let-7 leads to overexpression of HMGA2 in LMS.

miRNAs role was also investigated as potential diagnostic biomarkers in LMS. A group found that miR-221 levels could differentiate between LMS from their benign counterpart (leiomyoma) (Nuovo and Schmittgen, 2008). Another miRNA, miR-21 was differentially expressed in leiomyoma compared to LMS (Nuovo and Schmittgen, 2008). They demonstrated that miR-21 was and hormonally deregulated in leiomyomas. In addition to this study, Guled et al. demonstrated miRNAs including miR-199b-5p, miR-320a, miR-199a-3p, miR-126, and miR-22 were differentially expressed in LMS and undifferentiated pleomorphic sarcoma (UPS) as compared to mesenchymal stem cells (control) (Guled et al., 2014). These miRNAs indicate their importance in diagnosing and differentiating between LMS and UPS. Thus, further studies must be undertaken to functionally validate the miRNAs to confirm their clinical importance as diagnostic markers.

## Liposarcoma

Liposarcoma originates from fat cells and is the most common mesenchymal cancer. One of the miRNA studies was comprised of identifying miRNAs in liposarcomagenesis (Ugras et al., 2011). They performed small RNA sequencing between well-differentiated liposarcoma, dedifferentiated liposarcoma and normal adipose tissue samples. Such analysis revealed over 40 miRNAs including miR-21 and miR-26a, to be upregulated and miR-143 and miR-145 to be downregulated in dedifferentiated liposarcomas as compared to normal adipose tissue. They performed functional validation of miR-143 as a tumor suppressor as it represses expression of BCL2, topoisomerase 2A and PLK1 leading to cellular arrest. Similar independent studies revealed that miR-155 was overexpressed and miR-193b was downregulated in dedifferentiated liposarcoma as compared to their differentiated counterpart (Taylor et al., 2011b; Zhang et al., 2012). miR-155 targeted casein kinase 1 alpha that increased expression of beta-catenin signaling and cyclin D1, inducing cell growth and tumorigenesis (Zhang et al., 2012). The other study performed sequencing of genome, exome and transcriptome and found that miR-193b helps in distinguishing differentiated vs. dedifferentiated liposarcomas (Taylor et al., 2011b). Recently, a study demonstrated miR-143/145 and miR-144/451 cluster members were significantly downregulated in liposarcoma compared to adipocytes indicating their potential role as tumor suppressors (Gits et al., 2014). Over-expression of the clusters was able to significantly reduce proliferation and induce apoptosis in liposarcoma cell lines and hence these miRNAs can aid in diagnosis of liposarcoma patients.

## Other sarcoma types

There are other soft tissue sarcomas that are quite rare and understudied in relation to miRNAs.

Fibrosarcoma is derived from fibrous connective tissue. There are few studies that have investigated role of miRNAs in fibrosarcoma. miR-520c and miR-373 have been shown to activate Ras/MAPK pathway via activation of MMP9 in fibrosarcoma cell lines (Liu and Wilson, 2012). Another study revealed a potential tumor suppressor miRNA, miR-409-3p that downregulated angiogenin expression levels leading to cell death and tumor regression (Weng et al., 2012).

Angiosarcoma is another type of soft tissue sarcoma and our study identified miRNAs such as miR-552, −519, −515, −517a are upregulated in angiosarcomas as compared to other sarcomas (Sarver et al., 2010b). Further functional validation of these miRNAs will be required.

The other understudied soft tissue sarcoma in relation to miRNAs is hemangiosarcoma. There is only one study that demonstrates upregulation of miR-17-92 cluster in MYC-amplified hemangiosarcoma compared to hemangiosarcoma with no MYC amplification (Italiano et al., 2012). This implies that miR-17-92 overexpression is dependent on MYC amplification in this type of sarcoma. Also, upregulation of miR-17-92 decreased thrombospondin-1 (THBS1) expression that enhanced angiogenesis, indicating a MYC-miR-17-92 dependent pathogenesis in the MYC-amplified hemangiosarcoma tumor tissues.

## miRNA therapeutics - an insight on the future for sarcomas

**Figure 1:**
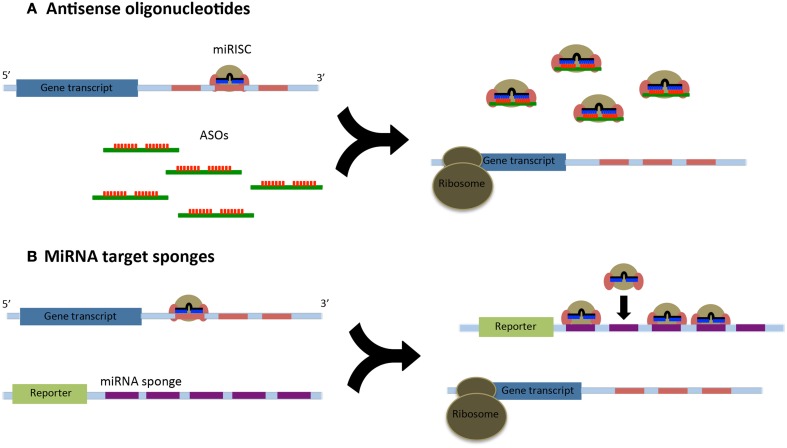
**(A)** Antisense oligonucleotides (ASOs) relieve the post-translational repression of target gene transcripts through their high affinity binding to the microRNA induced silencing complex (miRISC). Many ASOs are fully complementary to their miRNAs and will lead to their degradation. **(B)** MicroRNA sponges utilize a reporter construct with a 3′UTR containing multiple miRNA binding sites. These binding sequences act as decoys and can sequester a large number or miRISCs.

We have known about miRNAs and their role in mRNA regulation for over a decade; however, we still do not entirely understand the molecular mechanisms of how miRNAs regulate gene expression nor have we identified and established all the miRNA targets in the human genome (Fabbri et al., 2011). Thus, it becomes essential to understand the function and targets of miRNA by inhibiting the miRNA in a biological process. Such functional studies will also aid in targeting miRNAs that are deregulated in various sarcomas. To help understand the role of miRNAs in potential therapeutics, two strategies are commonly used: (i) direct cellular delivery of chemically synthesized miRNA inhibitors [such as antisense oligonucleotides (ASOs)] and (ii) delivery of a vector from which intracellular transcription of RNA inhibitors occurs (miRNA sponges or decoys) (Figure 1) (Li, 2014).

ASOs are short, single stranded DNA or RNA sequences that can specifically inhibit miRNAs (Dias and Stein, 2002). The mechanism of inhibition by ASOs is via Watson-Crick base pairing. The first ASOs were introduced via lipid-mediated transfection in *C. elegans* (Faltus et al., 2004; Zheng et al., 2010). Antagomirs or modified ASOs were the first miRNA inhibitors used in mammals (Krutzfeldt et al., 2005). Antagomirs contain a 2′-O-methyl modified ribose sugars terminal phosphorothiates and a cholesterol group at the 3′ end. The cholesterol group enhances the cellular uptake of the antagomirs due to their binding affinity to the cell surface membrane receptors. The challenge with antagomirs as a clinical application is that they require a higher dose to reach a certain efficacy as compared to other modified ASOs described below.

With the advancement in RNA chemistry, locked nucleic acid (LNA) was synthesized that has enhanced binding affinity toward miRNAs as compared to modified ASOs (Davis et al., 2006; Esau et al., 2006; Zheng et al., 2010). LNAs have 2′-4′-methylene bridge that locks the sugar ring in the 3′ endo conformation (Kumar et al., 1998). Such conformation along with addition of other modifications led to better affinity and stability of these ASOs *in vivo*. Subsequently, LNA modifications' application showed that it increased melting temperature and specificity in targeting miRNAs. The first known LNA-modified oligo was used to inhibit miR-122 that lowered plasma cholesterol levels in African green monkeys (Elmen et al., 2008). This non-human primate study showed that such oligonucleotides were specific, stable and non-toxic when administered intravenously for a prolonged period. Following this study, another group showed that there was inhibition of HCV replication via LNA that inhibited miR-122 in chimpanzees (Lanford et al., 2010). Interestingly, no viral escape was observed in the chimpanzees that were injected with LNA after 16 weeks, which was not obtained with antiviral therapy. After successful trials on non-human primates, LNAs against miR-122 are being tested in humans (Orom et al., 2006; Elmen et al., 2008). There has been a successful phase 1 trial that has paved its way to phase 2 study in human patients with chronic HCV infection (http://www.santaris.com).

As of today, such success has primarily been observed in liver infections. The reason is that the liver is amongst the most successful organs in the uptake of therapeutic RNAs. Not only is the bioavailability a challenge, but also there are other hurdles in therapeutic delivery of such RNAs that include size of the RNA and mode of delivery (Fabbri et al., 2011). Any RNAs less than 50 kDa are filtered through the kidney and excreted so local delivery of the modified RNAs is preferred. A possible solution to this challenge is to use PEGylated liposomes that are lipid vesicles within which nucleic acids can be encapsulated to avoid being filtered by the kidneys (Podesta and Kostarelos, 2009; McCaskill et al., 2013). To improve the mode of RNA delivery, one of the formulations used was lipid nanoparticle (Shi et al., 2011; Asai and Oku, 2014). The lipid nanoparticle consists of lipoplexes, which are cationic lipids that drive interaction between cationic lipids and the negatively charged siRNAs. One of the studies tested this delivery method in rodents and non-human primates and found the siRNA delivery that targets a hepatocyte mRNA (transthyretin) was effective at dose as low as 0.01 and 0.1 mg/kg, respectively (Semple et al., 2010). The mRNA levels were reduced to up to 30% after 48 h of administration. Another similar strategy with a more potent delivery formula called the lipidoid was devised, which contained lipids with amino polar head groups and non-polar alkyl tails (Love et al., 2010). This lipidoid, when administered in a similar fashion as the earlier study, decreased the transthyretin mRNA levels by 70% making it more effective than the lipoplex. Such advanced lipid-based delivery systems indicate that they will be useful in delivering ASOs to specific tissues in humans in the near future.

An alternative to ASOs is generating miRNA-binding RNA transcripts to sequester and inhibit specific miRNAs that can be expressed via viral vectors in cell lines, plants, or model animals (Ebert et al., 2007; Loya et al., 2009; Ebert and Sharp, 2010; Todesco et al., 2010; Fabbri et al., 2011). The first known miRNA decoy consisted of an adenoviral vector with miRNA-133 sites inserted in 3′UTR of GFP reporter gene under a RNA polymerase II promoter (Care et al., 2007). There are two commonly known miRNA decoys: miRZips and tough decoy RNAs (TuD RNAs), which are transcribed by RNA polymerase III (H1 or U6) (Haraguchi et al., 2009). miRZips are transcribed by RNA polymerase III H1 promoter and express a single miRNA decoy hairpin of which one strand is perfectly complementary to the mature miRNA. This blocks or “zips” the function of mature miRNA and hence the name miRZip. The TuDs are also transcribed by RNA polymerase III. It consists of a 60 bp long hairpin-shaped RNA which carries an internal loop that can be exposed to two miRNA binding sites with a central bulge (Androsavich and Chau, 2014). Such structural conformation helps in bypassing Ago2 mediated cleavage and RISC mediated destabilization eventually preventing its inactivation. This type of decoy provides longer-term inhibition of miRNAs than the miRZips or antisense RNA oligonucleotides. On the contrary, sponge miRNA inhibitors are RNA polymerase III transcribed non-structural RNA that harbors 4–12 consecutive miRNA binding sites with central bulges that can be stable integrated into the genome. Such sponges can be designed to be drug (tetracycline) inducible or can be under a tissue specific promoter (Faghihi et al., 2010; Stenvang et al., 2012; Cheng et al., 2013; Gebert et al., 2014). Such TuDs and sponges have been designed and shown to effectively inhibit expression of miR-21 and let-7 (Xie et al., 2012). Further advancement in the field would be to design such miRNA decoys that could inhibit specific miRNAs at a particular developmental stage or a specific tissue in an animal model system.

In addition to the mentioned “decoy” transcripts that compete with miRNAs and block their function, miRNA replacement therapy can be another approach to reintroduce missing miRNA/s. For instance, let-7 levels were found to be low in primary ovarian tumors and breast cell derived cancer stem cells indicating its role as tumor suppressor in certain cancers (Yu et al., 2007; Liu et al., 2013). Another commonly decreased level miRNA found in various cancers is miR-34 (Cheng et al., 2014; Okada et al., 2014). p53 is deleted in many cancer types and p53 promotes transcription of miR-34, hence its low expression. miR-34 also becomes an important tumor suppressor in many sarcoma types. For instance, miR-34 levels inversely correlate with poor patient survival outcomes indicating its potential role as a diagnostic marker in Ewing sarcoma (Marino et al., 2014). Re-introduction or replacement of such tumor suppressor miRNAs can be applied as targeted therapy or adjuvant therapy to the existing chemotherapy. Direct delivery of miRNAs or a viral vector encoding short hairpin RNAs that eventually are processed in mature miRNAs can be used to achieve the replenishment of the missing miRNAs (Kota et al., 2009; Bader et al., 2010). Multiple doses of miRNA with an optimal delivery method should be able to correct the miRNA imbalance in a cell type. Use of viral vectors can aid in better delivery, stability, and assessment of the miRNA/s being added to a cell or tissue type. In fact, miR-34 replacement therapy has shown promise against cancer cell survival, stemness, metastasis, and chemoresistance in various cancer cell types and animal models and is under Phase I clinical trial undertaken by MiRNA Therapeutics (Bader, 2012). Alternatively, viral vectors can be used to deliver specific gene target sequences to inhibit miRNAs in a specific organ, while restoring the function in the rest of the body (Santulli et al., 2014). For instance, adenoviral vector delivery of miR-126-3p target sequences at the 3′ end of exogenous *p27* selectively protects endothelial cells from p27 overexpression and also allows for complete restoration of endothelial cells (Santulli et al., 2014). However, such proof-of-concept need to be shown in cancer studies, where only the miRNA levels in tumors is affected without disrupting the miRNAs in the adjacent organs.

## Conclusion

We believe that understanding the miRNA-directed gene regulation will help us improve traditional gene therapy approaches. The reason is that a single miRNA has the ability to affect regulation of multiple genes and signaling pathways, and strategies to target such miRNAs can cease production of such deregulated genes. The anti-miRNAs and miRNA replacement therapeutic approaches unveil the importance and need for correction of the deregulated miRNAs and understand many new roles of individual miRNAs in various sarcoma types. Such a strategy would help develop drugs that are highly sensitive to multiple deregulated genes and will make better approval for the clinical trials. Moreover, many of the sarcomas are important pediatric cancers and such miRNA therapeutic approaches will help in improving and enhancing the survival rates of the pediatric patients. Nonetheless, efficient and targeted delivery, stability, and safety of such drugs still remain challenging but with the advancement of the miRNA field we believe that technologies will be developed to allow discovery and rapid development of effective miRNA therapeutics.

### Conflict of interest statement

The authors declare that the research was conducted in the absence of any commercial or financial relationships that could be construed as a potential conflict of interest.
